# Causal associations between insulin-like growth factor 1 and vitamin D levels: a two-sample bidirectional Mendelian randomization study

**DOI:** 10.3389/fnut.2023.1162442

**Published:** 2023-05-17

**Authors:** Zhaoyang Gou, Fan Li, Fengzhen Qiao, Gulinuer Maimaititusvn, Fang Liu

**Affiliations:** ^1^Department of Pediatrics, Shanghai East Hospital, Tongji University School of Medicine, Shanghai, China; ^2^Department of Pediatrics, Shanghai Tongji Hospital, Tongji University School of Medicine, Shanghai, China; ^3^Department of Gastroenterology, The First Hospital of Jilin University, Changchun, China

**Keywords:** insulin-like growth factor 1, serum 25-hydroxyvitamin D, vitamin D, Mendelian randomization, short stature

## Abstract

**Background:**

Insulin-like growth factor 1 (IGF-1) plays a vital role in the attainment and maintenance of bone mass throughout life and is closely related to the stature of children. 25-Hydroxyvitamin D (25-OHD) is an intermediate of vitamin D (Vit D) metabolism and a key indicator of Vit D nutritional status. Multiple studies have revealed that IGF-1 levels undergo a non-significant increase after Vit D supplementation. Here, we analyzed the causal and reverse causal relationships between 25-OHD and IGF-1 levels using Mendelian randomization (MR).

**Methods:**

Two-sample MR was used to estimate an unconfounded bidirectional causal relationship between the level of IGF-1 and those of Vit D and 25-OHD. Single nucleotide polymorphisms (SNPs) were filtered from genome-wide association studies (GWAS) after a comprehensive search of the Integrative Epidemiology Unit GWAS database. Several MR methods were employed, including inverse-variance weighted (IVW) method, and a sensitivity analysis was undertaken to detect whether pleiotropy or heterogeneity biased the MR results.

**Results:**

Genetically predicted IGF-1 was found to have a causal association with Vit D and serum 25-OHD levels, where Vit D and serum 25-OHD levels increased with increasing IGF-1 concentrations (Vit D: IVW β:0.021, 95% CI: 0.005–0.036, *p* = 7.74 × 10^–3^; 25-OHD: IVW β: 0.041, 95% CI: 0.026–0.057, *p* = 2.50 × 10^–7^). A reverse causal effect was also found, indicating Vit D and serum 25-OHD have a positive causal relationship with IGF-1 (Vit D: IVW β:0.182, 95% CI: 0.061–0.305, *p* = 3.25 × 10^–3^; 25-OHD: IVW β: 0.057, 95% CI = 0.017–0.096, *p* = 4.73 × 10^–3^). The sensitivity analysis showed that horizontal pleiotropy was unlikely to bias the causality in this study (MR-Egger: Vit D intercept *p* = 5.1 × 10^–5^, 25-OHD intercept *p* = 6.4 × 10^–4^ in forward analysis; Vit D intercept *p* = 6.6 × 10^–4^, 25-OHD intercept *p* = 1.9 × 10^–3^ in reverse analysis), and a leave-one-out analysis did not identify evidence of bias in the results.

**Conclusion:**

The results of the MR analysis provide evidence that IGF-1 has positive causal and reverse causal relationships with Vit D and serum 25-OHD, respectively, in European populations. Our findings also provide guidance for the prevention and treatment of short stature and other related diseases.

## 1. Introduction

Growth hormone (GH) produced in the pituitary gland stimulates the production of Insulin-like growth factor 1 (IGF-1) from the liver and other tissues (known as the GH–IGF1 axis) (1). The GH-IGF1 axis is an endocrine axis that plays an important role in children’s growth and development. The physiological actions of the GH–IGF1 axis on the skeleton are complex and derive from the dual effects of systemic and locally produced IGF1 as well as from the autocrine and paracrine actions of IGF binding proteins (IGFBPs) synthesized by bone cells. IGF-1, as the most abundant growth factor in the bone matrix, is an important regulator of bone remodeling and metabolism and plays an essential role in the attainment and maintenance of bone mass throughout life (1). *In vivo* studies using genetically modified mouse models have demonstrated that IGF-1 is both present in the circulation and locally expressed. Circulating IGF-1 is produced primarily by the liver under the stimulation of growth hormone (GH) and acts on osteoblasts (bone formation), osteoclasts (bone resorption), and other bone-related cells (1). IGF-1 can enhance bone resorption (2) and influence cortical bone integrity primarily by enhancing osteoblast differentiation, promoting the synthesis of receptor activators of the NF-κB ligand (RANKL), and preventing apoptosis. Moreover, IGF-1 is produced locally by osteoblasts under the stimulation of parathyroid hormone (PTH), which serves to maintain cancellous (or trabecular) bone integrity (3).

Some individuals with IGF-1 deficiency or IGF-1 overload have altered cortical bone and trabecular bone structure. In IGF-1 deficiency, anorexia nervosa (4), aging (5), and hypopituitarism (3), serum IGF-1 concentrations are reduced, leading to decreased bone weight or the slowing of growth due to a lack of physiologic effects of IGF-1 on osteoblasts. The response of osteoblasts to PTH may also be weakened. In acromegaly caused by GH-secreting pituitary adenoma, serum concentrations of IGF-1 and the PTH-dependent production of IGF-1 by osteoblasts are increased, with a consequent enhancement of bone remodeling. Bone resorption is also increased more than bone formation in this condition (1). GH–IGF axis dysfunction involves a variety of factors, such as reductions in both serum IGF-1 concentrations and the responsiveness of peripheral tissues to IGFs. When combined, these observations highlight that IGF-1 is an independent factor affecting physical growth.

In addition to its well-known role in promoting growth and metabolism, IGF-1 also improves lipid profile (6), lowers insulin levels, increases insulin sensitivity, and promotes glucose metabolism (7, 8). IGF-1 is also closely related to the occurrence and development of diseases such as diabetes, obesity, and metabolic syndrome. Furthermore, increasing evidence suggests that IGF-1 plays a special role in supporting the complex activities of cardiovascular (CV) function. IGF-1 promotes heart development, increases cardiac output, stroke volume, contractility, and ejection fraction. Human studies have also shown that low levels of serum free or total IGF-1 are associated with an increased risk of cardiovascular and cerebrovascular diseases (9). The role of IGF-1 in the body is becoming increasingly apparent.

Vitamin D (Vit D) is a fat-soluble vitamin synthesized by the liver whose main biological function is to regulate the absorption of calcium and phosphorus and the mineralization of bones. Vit D deficiency can hinder bone matrix synthesis by osteoblasts and the mineralization of collagen fibers, thereby affecting bone growth, formation, and absorption in children (10). Vit D is absorbed via the intestine and is transported to the liver through the circulation, where it is converted to 25-hydroxyvitamin D (25-OHD) (11). Compared with Vit D, 25-OHD is present at higher concentrations in serum, is more stable, and has a longer half-life, which renders it an optimal biomarker for the nutritional status of Vit D. 25-OHD is a steroid pro-hormone and a fat-soluble metabolite of cholecalciferol, a form of Vit D, which is predominately synthesized following exposure to ultraviolet light or obtained from dietary sources (12). 25-OHD plays an important role in regulating calcium and phosphorus concentrations; influences cell proliferation, differentiation, and apoptosis, and has immune-modulating properties (13).

Children with short stature are also commonly associated with vitamin D deficiency, and the decreases in calcium and phosphorus absorption, bone formation, and bone resorption caused by Vit D deficiency are also related to children’s restricted height and weight gain (14). Serum 25-OHD, IGF-1 level has high sensitivity and specificity for the diagnosis of children with idiopathic short stature, which is positively correlated with height and body weight. Research has shown that GH induces liver synthesis of IGF-1 during childhood growth and development, increasing P450 activity in liver mitochondria, whereas P450 directly induces 25-hydroxylation of Vit D and increases sufficient serum concentration; normal bone mineralization is also affected, resulting in abnormal bone metabolism in children (15). The lack of IGF-1 may also lead to poor height development, abnormally low body weight, low BMI, and other adverse conditions. Therefore, IGF-1 serum concentration has a good predictive value for the Subsequent treatment and follow-up of children with ISS. In clinical work, the growth and development of children can be evaluated by measuring serum 25-OHD and IGF-1 levels. However, whether a causal relationship exists between the two factors remains unclear, and whether vitamin supplementation can improve bone density status and IGF-1 levels in children remains controversial (16).

Traditional research methods are divided into observational and experimental research. The former can only be performed under natural conditions, and no artificial intervention measures can be included (17); it is also subject to reverse causal associations, with many potential confounding factors. Although experimental research can make up for many of the limitations of observational research, experimental design and implementation must be both precisely specified and strictly controlled, rendering it difficult to implement. Because the implementation of experimental research must consider ethics, it is often difficult to use this method to directly study the etiology of diseases (18). Mendelian randomization (MR) has been proposed as a method for overcoming these limitations. According to Mendelian inheritance, the alleles of parents are randomly transmitted to their offspring, which is equivalent to the random grouping process used in randomized controlled trials (17), and the whole process is not affected by confounding factors such as environmental exposure, social status, and behavior (19). Furthermore, MR is a good fit for chronological plausibility because genetic variation is inherited and remains relatively stable (17). Therefore, in this study, we used MR to explore whether a causal or reverse causal relationship exists between IGF-1 with Vit D and 25-OHD.

## 2. Materials and methods

### 2.1. Study design

**FIGURE 1:**
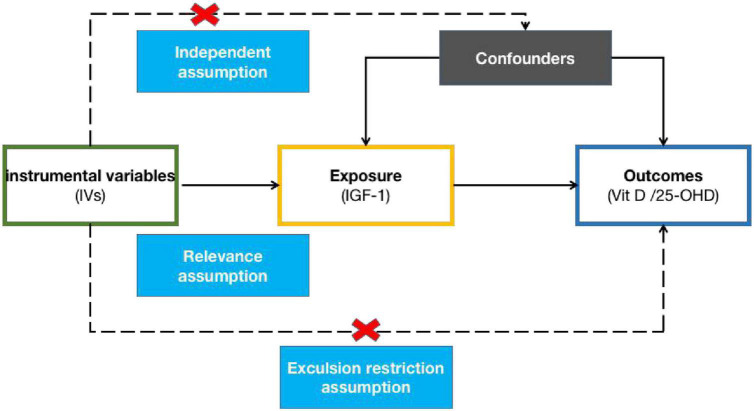
A directed acyclic graph consisting of the genetic instrument, exposure, and outcome.

To perform MR analysis, the instrumental variables (IVs) must satisfy the following assumptions: First, the IVs must be significantly correlated with risk factors (relevance assumption); second, they must not be related to any confounding factor (independence assumption); and third, they must only indirectly affect outcomes through risk factors (exclusion restriction assumption). A directed acyclic graph (Figure 1) was constructed using single nucleotide polymorphisms (SNPs), risk factors (IGF-1), and outcomes (Vit D and 25-OHD) to illustrate the basic assumptions of the MR analysis. Among them, the allele frequencies of the same SNPs may differ according to the ancestral population, and ancestors may be associated with certain risk factors. Therefore, to minimize population stratification, only samples from European subjects were included in this MR study.

### 2.2. Exposure data selection

Appropriate genetic variants that could serve as valid IVs for exposure and outcomes were selected from the publicly available Integrative Epidemiology Unit (IEU) genome-wide association studies (GWAS) database (20). SNPs were selected as IVs for exposure (IGF-1) from the IEU GWAS database using kinship relatedness, predicted ancestry, principal components analysis, and per-SNP INFO scores provided by the UK Biobank^1^ genetics team. Data for 487,409 individuals were filtered down to that for 337,199 individuals (Table 1). SNPs with a minor allele frequency (MAF) of >0.1% and a Hardy–Weinberg equilibrium (HWE) *p*-value of >1 × 10^–10^ were included. Finally, 10.8 million SNPs remained for analysis.

**TABLE 1:** Characteristics of the study population.

Phenotype	SNPs available	Sample size	References	Population
IGF-1	13,586,000	337,199	(48)	European
Vitamin D	2,538,249	79,366	(21)	European
Serum 25-OHD	8,644,129	417,580	(22)	European

SNP, small nucleotide polymorphism; 25-OHD, 25-hydroxyvitamin D.

### 2.3. Outcome data sources

As for the exposure data, Vit D levels were obtained from Jiang et al., who undertook a large, multicenter, genome-wide association study of 31 cohorts in Europe, Canada, and the US. They then discovered that the meta-analysis consisted of 79,366 samples of European descent drawn from 31 epidemiological cohorts (21). The largest GWAS was selected, which comprised a GWAS of serum 25-OHD levels in a large population cohort that contained phenotypic, genotypic, and clinical information for more than 502,000 individuals (age range: 40–69 years) (Table 1). Data regarding Vit D and 25-OHD levels (data field 30890) were available for 449,978 of the 502,536 participants (90%), which were mostly (448,376; 99.6%) obtained during the initial assessment visit (22). The UK Biobank data were limited to 417,580 individuals of European ancestry for whom 25-OHD concentrations were available; 318,851 of the 417,580 individuals were unrelated. The UK Biobank extracted variants with a minor allele count (MAC) of >5 and an imputation score of >0.3 for all individuals and converted genotype probabilities to hard-call genotypes using PLINK2 (–hard-call 0.1) (23). Variants with a genotype missingness of >0.05, an HWE *p*-value of >1 × 10^–5^, and an MAF of <0.01 were excluded. European ancestry was assigned based on a >0.9 posterior probability of belonging to the 1KGP European reference cluster (22).

### 2.4. SNP selection

A set of quality control steps was performed to select qualified SNPs from the GWAS summary results. In the pooled statistics, SNPs with genome-wide association (*p* < 5 × 10^–8^), independent inheritance (*r*^2^ < 0.001), and no linkage disequilibrium (LD, kb = 10,000) were selected (24). Data for exposure and outcomes were pooled using SNPs screened for these conditions, yielding the data for MR analysis. Palindromic SNPs (those with A/T or G/C alleles) with medium gene frequency (0.01< allele frequency <0.30) were excluded from the instrument SNPs mentioned above. SNPs with an MAF of <0.01 were also excluded. The *F*-statistic of the SNPs was also calculated to measure the intensity of the instruments. SNPs with an *F*-statistic of <10 were excluded and are usually labeled as “weak tools” (25). These strictly selected SNPs were used for subsequent MR analysis. The proportion of phenotypic variation explained by the SNPs was estimated.

### 2.5. Effect size estimate

In the MR analysis, inverse-variance weighted (IVW), MR-Egger, and weighted median (WM) methods were primarily employed. The WM method assesses the relationship between exposure and outcome with a single SNP weighted for the precision of the MR estimates and offers consistent estimates (26). IVW, the most frequently used method for MR analysis, uses a meta-analysis approach to combine SNP ratio estimates in an inverse-variance weighted manner, and obtains an estimate of the effect of risk factors on outcomes (24) characterized by the fact that the existence of intercepts is not considered at the time of regression. Meanwhile, MR-Egger regression creates a weighted linear regression of the outcome coefficients on the exposure coefficients and can provide unbiased estimates even when all genetic variants are invalid (27, 28). MR-Egger regression is the preferred method when there is evidence of pleiotropy. *I*^2^ values were also calculated to quantify the extent to which the NO Measurement Error (NOME) assumption was violated by MR-Egger. The results were corrected when *I*^2^ < 90% (29). In addition, simple-mode and weighted-mode methods were also used for calculation.

The principles of two-sample MR were applied to assess the role of exposure (IGF-1) in the susceptibility of the outcomes (Vit D and 25-OHD). The SNPs were selected as the IVs according to the criteria listed above, and the effects of the selected SNPs on the exposure and outcomes were estimated.

### 2.6. Sensitivity analyses

To exclude possible violations of the MR assumptions, multiple sensitivity analyses were conducted to determine whether heterogeneity and pleiotropy within the tested genetic instruments could bias the MR results. Sensitivity testing is mainly carried out based on heterogeneity test, pleiotropy test, and Leave-one-out sensitivity analysis.

The heterogeneity test is mainly used to test the difference between individual SNPs. Both the MR-Egger and IVW methods were used for the determination of heterogeneity; *Q*-statistic values of *p* < 0.05 and heterogeneity test-*I*^2^ (het-I^2^) > 40% indicated significant heterogeneity for the IVW method.

The pleiotropy test is primarily employed to detect the presence of horizontal pleiotropy in multiple SNPs. Pleiotropy refers to the phenomenon in which a single locus affects multiple phenotypes. Horizontal pleiotropy arises when a genetic variant is associated with more than one phenotype in separate pathways, which can invalidate the results of MR analyses. The MR-Egger regression intercept was evaluated and the MR-PRESSO global test (30) was conducted to estimate the presence of pleiotropy; in the former, the presence of pleiotropy is usually established when the MR-Egger intercept term differs from 0, and a large difference indicates the existence of pleiotropy between SNPs. MR-PRESSO is an extension of previous approaches that utilize the general model of multi-instrument MR on summary statistics and is best used to identify inconsistencies between the genetic associations of different genetic variants and remove outlying genetic variants. This bidirectional MR analysis showed that there was no horizontal pleiotropy between SNPs (*p* > 0.05).

Leave-one-out sensitivity analysis is implemented by removing a different SNP in each iteration when performing the MR analysis to guarantee that the MR estimates are not influenced by the inclusion of proxy SNPs. However, if all the lines are on the same side of 0 in the leave-one-out plot, this indicates that, irrespective of which SNP is removed, the result will not be fundamentally affected, that is, the result is robust.

All statistical tests were performed using the “Two Sample MR” package for R version 0.5.6. The “two sample MR” codes from our study are available at https://gwas.mrcieu.ac.uk.

## 3. Results

### 3.1. SNP validation

We integrated 364 SNPs displaying genome-wide association (*p* < 5 × 10^–8^) and independent inheritance with no LD (*r*^2^ < 0.001, kb = 10,000) as IV SNPs for IGF-1 after removing one (rs2925656) incompatible allele from IGF-1 SNPs associated with 25-OHD. However, 29 (rs11208537, rs1150781, rs11757793, rs12051698, rs125124, rs13232120, rs13237149, rs1351893, rs1759 7773, rs1782652, rs1994147, rs2274224, rs2716929, rs273951, rs35713203, rs3858525, rs4782568, rs539687, rs55843942, rs5755 943, rs6822348, rs6913063, rs7012213, rs72884378, rs7662792, rs7952602, rs8055075, rs9886703, rs9896243) and 8 (rs10657263, rs11757793, rs11967262, rs2274224, rs3858525, rs4466086, rs4782568, and rs76265107) IGF-1 SNPs associated with Vit D and 25-OHD, respectively, that were palindromic and displayed intermediate allele frequencies were excluded. We carried out a series of quality-control steps to select eligible SNPs from the GWAS summary results, yielding the data for MR analysis. Then, SNPs with horizontal pleiotropy were identified and excluded using MR-PRESSO; a total of 106 and 31 SNPs associated with Vit D and 25-OHD, respectively, were separately removed (Table 2).

**TABLE 2:** The effect estimates of the Mendelian randomization (MR) analysis.

Exposure	Outcome	MR method	β	se	Lo-CI	Up-CI	*p*-value
IGF-1	Vitamin D	MR-Egger	0.018999812	0.01520897	−0.010809769	0.048809393	0.213195575
		Weighted median	0.031096407	0.011584079	0.008391612	0.053801201	0.007265815
		Inverse-variance weighted	0.020621847	0.007743419	0.005444747	0.035798947	0.007741403
		Simple mode	0.020899676	0.028156206	−0.034286488	0.07608584	0.458882307
		Weighted mode	0.027161608	0.012425803	0.002807034	0.051516182	0.030106294
Vitamin D	IGF-1	MR-Egger	0.157114122	0.232955357	−0.299478379	0.613706622	0.537003558
		Weighted median	0.144360498	0.056881533	0.032872694	0.255848302	0.011151505
		Inverse-variance weighted	0.182848013	0.062131121	0.061071015	0.30462501	0.003251139
		Simple mode	0.133477074	0.092006864	−0.04685638	0.313810528	0.206555075
		Weighted mode	0.143187284	0.069061923	0.007825915	0.278548654	0.092847198
IGF-1	Serum 25-hydroxyvitamin D	MR-Egger	0.021265266	0.015677295	−0.009462233	0.051992765	0.176011551
		Weighted median	0.025650252	0.010171023	0.005715047	0.045585457	0.011672456
		Inverse-variance weighted	0.041381371	0.008023283	0.025655736	0.057107005	2.50054 × 10^–7^
		Simple mode	0.06860863	0.029507856	0.010773232	0.126444029	0.02075211
		Weighted mode	0.028706186	0.012075818	0.005037584	0.052374789	0.018090546
Serum 25-hydroxyvitamin D	IGF-1	MR-Egger	0.004682825	0.032548237	−0.059111719	0.068477369	0.885957799
		Weighted median	0.035994931	0.018326928	7.41526E–05	0.071915709	0.049524746
		Inverse-variance weighted	0.056769621	0.020097767	0.017377997	0.096161245	0.004732889
		Simple mode	−0.016451297	0.044220901	−0.103124263	0.070221669	0.710833648
		Weighted mode	0.025334079	0.015999703	−0.006025339	0.056693498	0.117177654

The *F*-values were calculated for the remaining 187 and 302 SNPs to measure the strength of the instruments. SNPs with an *F*-statistic of <10 are normally labeled as “weak instruments” (25). In this study, the *F*-statistic values for all the SNPs were >10, indicating that there were no weak IVs. Five and nine ambiguous SNPs associated with Vit D and 25-OHD, respectively, were then dropped during the analysis, and the causal direction of the remaining SNPs was detected and found to be positive. These results indicated that the variables satisfied the strong relevance assumption of MR and that the instrument bias was weak and could not substantially influence the estimations of causal effects (Table 2).

**FIGURE 2:**
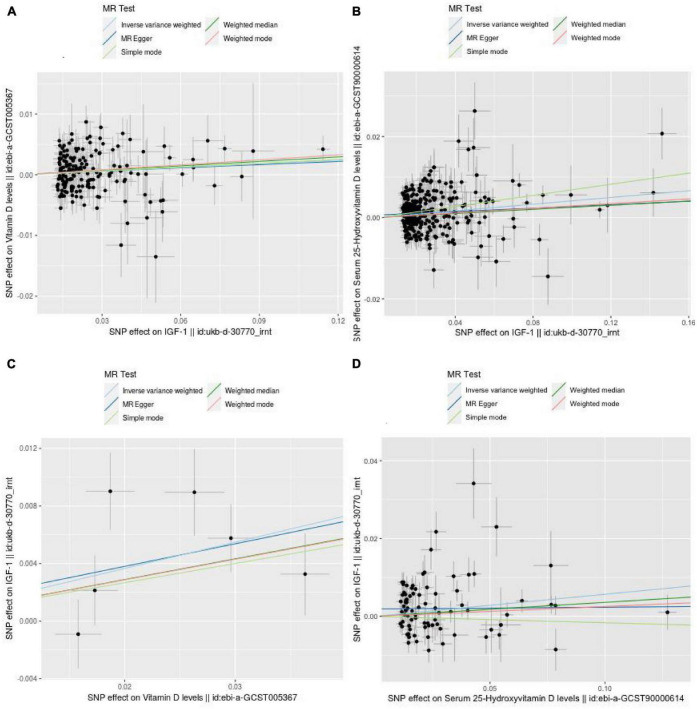
Scatter plots showing the causal effect of exposure on outcome. **(A)** The effect of IGF-1 on vitamin D. **(B)** The effect of IGF-1 on 25-hydroxyvitamin D. **(C)** The effect of vitamin D on IGF-1. **(D)** The effect of 25-hydroxyvitamin D on IGF-1.

Finally, 182 and 293 SNPs were screened to determine the causal relationship between IGF-1 and Vit D or 25-OHD levels, respectively. The distribution of the influence of individual SNPs on the results can be seen in the scatter plots in Figure 2.

### 3.2. Main results

The results obtained using the five MR methods (IVW, WM, MR-Egger, Simple Mode, and Weighted Mode) were generally consistent and positive. Tables 2, 3 show all MR analysis and sensitivity analysis assessments.

**TABLE 3:** Tests of MR-Steiger causal direction, MR-Egger *I*^2^, heterogeneity, and pleiotropy.

Exposure	Outcome	Heterogeneity test				MR-Egger intercept	*p*-value	MR-Steiger causal direction	MR-Egger *I*^2^
		**MR method**	***Q***	***Q*_*p*-value**	***I* ^2^**				
IGF-1	Vitamin D	MR-Egger	251.7	3.4 × 10^–4^	0.28	5.1 × 10^–5^	0.90	True	0.99
		Inverse-variance weighted	251.7	4.0 × 10^–4^	0.28				
Vitamin D	IGF-1	MR-Egger	10.3	3.5 × 10^–2^	0.61	6.6 × 10^–4^	0.91	True	0.99
		Inverse-variance weighted	10.4	6.6 × 10^–2^	0.52				
IGF-1	Serum 25-OHD	MR-Egger	694.5	5.3 × 10^–35^	0.58	6.4 × 10^–4^	0.14	True	0.99
		Inverse-variance weighted	699.8	1.7 × 10^–35^	0.58				
Serum 25-OHD	IGF-1	MR-Egger	245.7	1.7 × 10^–18^	0.67	1.9 × 10^–3^	0.05	True	0.99
		Inverse-variance weighted	258.0	4.4 × 10^–20^	0.68				

25-OHD, 25-hydroxyvitamin D; MR, Mendelian randomization.

The IVW and WM MR results showed that a significant causal relationship (*p* < 0.05) existed between IGF-1 levels and Vit D/serum 25-OHD levels, and there was also a positive correlation between them (Vit D: IVW β: 0.021, 95% CI: 0.005–0.036, *p* = 7.74 × 10^–3^; Weighted Median β: 0.031, 95% CI: 0.008–0.054, *p* = 7.26 × 10^–3^; Weighted Mode β: 0.027, 95% CI: 0.003–0.052, *p* = 3.01 × 10^–2^; 25-OHD: IVW β: 0.041, 95% CI: 0.026–0.057, *p* = 2.50 × 10^–7^; Weighted Median β: 0.026, 95% CI: 0.006–0.046, *p* = 1.17 × 10^–2^; Simple Mode β: 0.069, 95% CI: 0.011–0.126, *p* = 2.07 × 10^–2^; Weighted Mode β: 0.029, 95% CI: 0.005 to 0.052, *p* = 1.81 × 10^–2^). However, when the MR-Egger regression method and Simple Mode were used, no significant association was detected between IGF-1 and Vit D levels (MR-Egger β: 0.019, 95% CI: −0.011 to 0.049, *p* = 0.21; Simple Mode β: 0.021, 95% CI: −0.034 to 0.076, *p* = 0.459) or between IGF-1 and 25-OHD (MR-Egger β: 0.021, 95% CI: −0.009 to 0.052, *p* = 0.176) levels when the MR-Egger regression method was used (Table 2).

MR-Egger regression was used to assess pleiotropy, with the results showing that horizontal pleiotropy was unlikely to bias the causal relationship between IGF-1 and Vit D (*p* = 0.90) or 25-OHD (*p* = 0.14) (Table 3). The IVW estimates were consistent with the WM estimates because pleiotropy assessments are considered not pleiotropic between these SNPs and the causality of IVW estimates is more robust than that of MR-Egger regression estimates because results considering GWAS are phenotypically normalized (1). The relationship between Vit D and IGF-1 levels was consistent with that between serum 25-OHD and IGF-1 levels, thus rendering the results more reliable. These results strongly suggested that both Vit D and serum 25-OHD levels increase with increasing IGF-1 levels.

### 3.3. Sensitivity analysis

**FIGURE 3:**
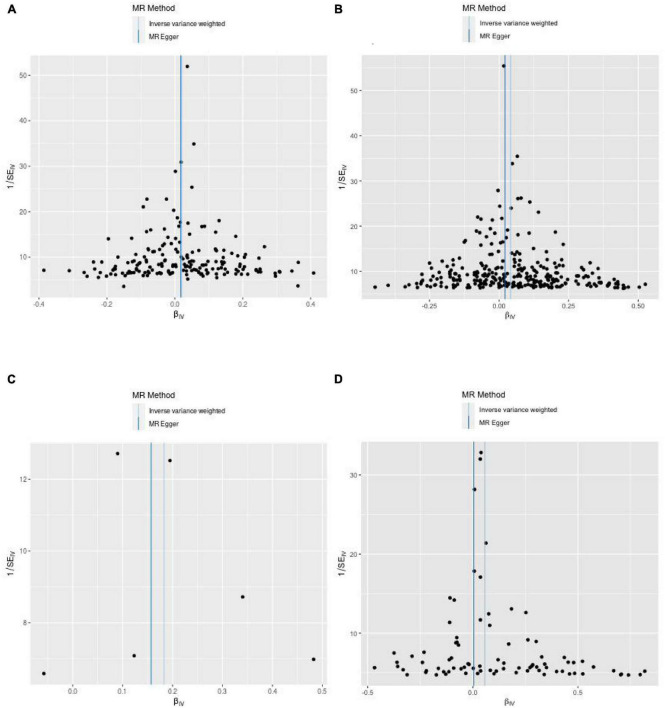
Funnel plots from exposure on outcome. **(A)** From IGF-1 on vitamin D. **(B)** From IGF-1 on 25-hydroxyvitamin D. **(C)** From vitamin D on IGF-1. **(D)** From 25-hydroxyvitamin D on IGF-1.

(1) Between estimates of SNPs for IVW assays, MR-Egger, Cochran *Q*-values, and het-I^2^ indicate significant heterogeneity (26) (Vit D: MR-Egger *Q* = 251.7, het-I^2^<0.4, *p* < 0.05; IVW *Q* = 251.7 het-I^2^<0.4, *p* < 0.05; 25-OHD: MR-Egger *Q* = 694.5, het-I^2^ > 0.4, *p* < 0.05; IVW *Q* = 699.8, het-I^2^ > 0.4, *p* < 0.05) (Table 3). In this case, a random-effects model was used (31). (2) After we used the MR-PRESSO test to exclude SNPs with horizontal pleiotropy, the MR-Steiger results indicated that there was no reverse in any of the selected SNPs, thus supporting a causal relationship between IGF-1 levels and those of Vit D and its metabolic intermediate, 25-OHD (Table 3). (3) The leave-one-out analysis also showed that none of the SNPs drove MR estimates, which meant that the results were robust (Supplementary Figure 1). Furthermore, a funnel plot (Figure 3) was used to test the InSIDE assumption of the MR-Egger method, which was satisfied, as the left and right sides of the funnel plot were symmetrical (27). At the same time, the *I*^2^ values of MR-Egger in this study were all >99%, indicating that the conclusions will not be affected by outliers and strong influence points.

### 3.4. Bidirectional MR

We also explored whether Vit D and serum 25-OHD levels affect the concentrations of IGF-1. For this, we reversed the function of exposures and outcome to perform a bidirectional MR analysis and determine the impact of increased IGF-1 serum levels. We selected SNPs carefully and strictly from the IEU GWAS database. We then used the corresponding effect estimates from the IEU GWAS database as results (1) and applied the same MR method as above. To explore the causal effects of Vit D and 25-OHD levels on IGF-1 levels, we selected 6 and 83 significant and independent SNPs, respectively, retrieved from the IEU GWAS database (Table 4).

**TABLE 4:** The numbers of excluded single nucleotide polymorphisms (SNPs) and identified instrumental SNPs.

Exposure	Outcomes	Missing outcome	Weak IV	Pleiotropy	Leave-one-out	Palindromic structure	Incompatible alleles	Wrong casual direction	Ambiguous SNPs	Eligible SNPs
IGF-1	Vitamin D	42	0	106	0	29	0	0	5	182
Vitamin D	IGF-1	0	0	0	0	2	0	0	0	6
IGF-1	Serum 25-OHD	31	0	31	0	8	1	0	9	293
Serum 25-OHD	IGF-1	0	0	27	0	1	0	0	1	83

IV, instrumental variables; 25-OHD, 25-hydroxyvitamin D.

Overall, all selected tools displayed *F*-statistic values of >10. We found important evidence for the existence of a causal relationship between increased levels of Vit D and 25-OHD, and IGF-1 risk, based on IVW and WM regression methods (Vit D: IVW β: 0.183, 95% CI: 0.061–0.305, *p* = 3.25 × 10^–3^; Weighted Median β: 0.144, 95%CI: 0.033–0.256, *p* = 1.12 × 10^–2^; 25-OHD: IVW β: 0.057, 95% CI: 0.017–0.096, *p* = 4.73 × 10^–3^; Weighted Median β: 0.036, 95% CI: 7.42 × 10^–5^ to 0.072, *p* = 4.95 × 10^–2^). When the MR-Egger, Simple Mode, and Weighted Mode regression methods were used, no significant association was found between Vit D and IGF-1 (MR-Egger β: 0.157, 95% CI: −0.299 to 0.614, *p* = 0.537; Simple Mode β: 0.133, 95% CI: −0.047 to 0.314, *p* = 0.207; Weighted Mode β: 0.143, 95% CI: 0.008–0.279, *p* = 9.28 × 10^–2^) or between 25-OHD and IGF-1 (MR-Egger β: 0.005, 95% CI: −0.059 to 0.068, *p* = 0.886; Simple Mode β: −0.016, 95% CI: −0.103 to 0.070, *p* = 0.711; Weighted mode β: 0.025, 95% CI: −0.006 to 0.057, *p* = 0.117) (Table 2). The MR-PRESSO test results were consistent with those obtained using IVW, WM, and MR-Egger regression. When we used the MR-PRESSO global test, we removed 0 and 27 SNPs of the horizontal pleiotropy of Vit D and 25-OHD on IGF-1 risk, respectively (Table 4). We performed an MR-Egger regression to assess pleiotropy, with the results showing that horizontal pleiotropy was unlikely to exist for the causal relationship between Vit D (*p* = 0.91) and IGF-1 (Table 3). However, the presence of horizontal pleiotropy could affect the causal relationship between 25-OHD (*p* = 0.05) and IGF-1 (Table 3). The leave-one-out analysis also revealed that no single SNP drove the MR estimates, indicating that the result was robust (Supplementary Figure 1). Cochran Q and het-I^2^ also indicated that heterogeneity existed among IV estimates determined using IVW, MR-Egger, and maximum likelihood methods (Table 3). The funnel plots showed the extent of heterogeneity among the SNP estimates.

In conclusion, we found a positive causal association between IGF-1 and serum 25-OHD level by IVW and WM methods. The statistical test for bidirectional MR analysis was bilateral, and the results of the MR and sensitivity analyses on the causal effect of serum 25-OHD levels on IGF-1 levels were significant (IVW *p* < 0.0047).

## 4. Discussion

To our knowledge, this is the first time that a large GWAS database and MR analysis have been used to investigate whether a causal relationship exists between IGF-1 and serum Vit D and 25-OHD levels. We found causal and reverse causal relationships between the concentrations of IGF-1 and those of Vit D and serum 25-OHD levels in people of European ancestry, respectively. Additionally, the results were robust, which ensured that our study met the relevance and independence assumptions. In addition, we performed sensitivity analyses to ensure that the exclusion restriction assumption was held.

Insulin-like growth factor 1 has been shown to stimulate the production of 1-α-hydroxylase, which promotes Vit D production in the kidneys (32). Additionally, an *in vitro* study reported that a high number of Vit D receptors in liver and pituitary cells, which are the primary means for IGF-1 and GH secretion, can explain why Vit D can exert positive effects on IGF-1 concentrations (33). In mouse models, knocking out the Vit D receptor (VDR) has been found to reduce IGF-1 levels (34). In humans, some observational studies have shown a correlation between IGF-1 concentrations in serum and Vit D levels (35–37). Some studies suggest that Vit D can stimulate IGF-1 production in the liver (38). Therefore, it has been suggested that Vit D supplementation can affect IGF-1 levels (16, 39). However, in one quantitative meta-analysis (40) of five studies (eight arms) containing 841 participants (41–45), no significant increase in overall IGF-1 was reported after supplementation with Vit D, but there was significant heterogeneity. Thus, whether a causal relationship exists between the two factors remains unclear. The sample size included in this analysis was small, and the sampling error and ethnic differences were large. Furthermore, the research process is inevitably affected by confounding factors such as environmental exposure, social status, and behavior (e.g., light exposure, season, and family environment) and is easily disturbed when exploring causality and reverse causality.

Fortunately, MR makes up for the abovementioned shortcomings very well. The results of the MR analysis show that IGF-1 has positive causal and reverse causal relationships with Vit D and serum 25-OHD, respectively, in European populations, which is contrary to previous studies where Vit D and serum 25-OHD levels increased with increasing IGF-1 concentrations. Moreover, Vit D and serum 25-OHD have a positive causal relationship with IGF-1. In this study, horizontal pleiotropy was unlikely to bias the causality based on the sensitivity analysis. The leave-one-out analysis did not identify evidence of bias in the results. There was also some heterogeneity among the results of this study, and the statistical power of our conclusions was calculated using network power calculators^2^ [Burgess (46)], which was very good. Results were considered statistically significant when *p* < 0.05. Our study supported causal and reverse causal relationships between Vit D and serum 25-OHD, respectively, and the results were robust because of the large sample size and the use of multiple sensitivity analyses in the GWAS study populations.

The level of IGF-1 is positively correlated with height and weight and has a protective effect on the cardiovascular system. It can also improve blood lipid levels and insulin resistance. Therefore, the IGF-1 level has certain clinical predictive value in diseases such as dwarfism, cardiovascular disease, cerebrovascular disease, obesity, and type 2 diabetes. The concentration of IGF-1 increases with the increase of vitamin D and plasma 25-OHD levels, and vitamin D supplementation may have some significance in the treatment of the above-mentioned diseases.

Despite its many advantages, there were some shortcomings in this study. First, we did not explore the effect of IGF-1 or Vit D supplementation on the corresponding outcomes due to data limitations. Second, Considering the gender-specific correlation of IGF-1 stimulated by growth hormone (47), gender-specific MR analysis should be conducted. However, we have not yet found gender-stratified data suitable for MR analysis. Therefore, in future studies, further gender-stratified exploration of GWAS data should be carried out, and corresponding exploration should be conducted in terms of mediation MR or other aspects. Third, the ratio estimation method used in this study was based on the existence of a linear relationship between exposure and results, and the possibility of a non-linear relationship between the two cannot be excluded; analysis in conjunction with clinical practice is required to assess this possibility. At last, despite greatly reducing the impact of population stratification on MR analysis, both the exposure and outcome data in our study were derived from a European cohort.

Future research directions can focus on the effects of IGF-1 and Vit D supplementation at different doses and times on Vit D levels and IGF-1 levels, and their mechanisms, aiming to find the pathway of the two effects to better guide the clinical supplementation of Vit D and growth hormones and find a suitable balance. The effects of supplementation on children’s height and bone density should also be explored, as well as any corresponding side effects. Finally, future studies could be conducted in populations other than those of European ancestry.

## Data availability statement

The original contributions presented in this study are included in the article/Supplementary material, further inquiries can be directed to the corresponding author.

## Author contributions

ZG and FQ conceived and designed the study. ZG and FLi provided the “TwoSampleMR” package codes in R language and analyzed the data. ZG, GM, and FLi drafted the manuscript. FLiu and FQ gave constructive suggestions during the writing of the manuscript. All authors read and approved the final version of the manuscript.

## Footnotes

1
www.nealelab.is/uk-biobank

2
https://sb452.shinyapps.io/power/

## References

[1] Mazziotti G Lania AG Canalis E. Skeletal disorders associated with the growth hormone-insulin-like growth factor 1 axis. *Nat Rev Endocrinol.* (2022) 18:353–65. 10.1038/s41574-022-00649-8 35288658

[2] Lindsey R Mohan S. Skeletal effects of growth hormone and insulin-like growth factor-I therapy. *Mol Cell Endocrinol.* (2016) 432:44–55. 10.1016/j.mce.2015.09.017 26408965 PMC4808510

[3] Mazziotti G Frara S Giustina A. Pituitary diseases and bone. *Endocr Rev.* (2018) 39:440–88. 10.1210/er.2018-00005 29684108

[4] Schorr M Miller KK. The endocrine manifestations of anorexia nervosa: mechanisms and management. *Nat Rev Endocrinol.* (2017) 13:174–86. 10.1038/nrendo.2016.175 27811940 PMC5998335

[5] Van Varsseveld NC Sohl E Drent ML Lips P. Gender-specific associations of serum insulin-like growth factor-1 with bone health and fractures in older persons. *J Clin Endocrinol Metab.* (2015) 100:4272–81. 10.1210/jc.2015-2549 26323023

[6] Mauras N Martinez V Rini A Guevara-Aguirre J. Recombinant human insulin-like growth factor I has significant anabolic effects in adults with growth hormone receptor deficiency: studies on protein, glucose, and lipid metabolism. *J Clin Endocrinol Metab.* (2000) 85:3036–42. 10.1210/jcem.85.9.6772 10999782

[7] Sesti G Sciacqua A Cardellini M Marini M Maio R Vatrano M Plasma concentration of IGF-I is independently associated with insulin sensitivity in subjects with different degrees of glucose tolerance. *Diabetes Care.* (2005) 28:120–5. 10.2337/diacare 15616244

[8] Sjögren K Wallenius K Liu J Bohlooly Y Pacini G Svensson L Liver-derived IGF-I is of importance for normal carbohydrate and lipid metabolism. *Diabetes.* (2001) 50:1539–45. 10.2337/diabetes.50.7.1539 11423474

[9] Macvanin M Gluvic Z Radovanovic J Essack M Gao X Isenovic E. New insights on the cardiovascular effects of IGF-1. *Front Endocrinol.* (2023) 14:1142644. 10.3389/fendo.2023.1142644 36843588 PMC9947133

[10] Kord-Varkaneh H Rinaldi G Hekmatdoost A Fatahi S Tan SC Shadnoush M The influence of vitamin D supplementation on IGF-1 levels in humans: a systematic review and meta-analysis. *Ageing Res Rev.* (2020) 57:100996. 10.1016/j.arr.2019.100996 31816443

[11] Christakos S Dhawan P Porta A Mady J Seth T. Vitamin D and intestinal calcium absorption. *Mol Cell Endocrinol.* (2011) 347:25–9. 10.1016/j.mce.2011.05.038 21664413 PMC3405161

[12] Xie Z Cheng Q Ding Y. Vitamin D metabolism and effects. *Chin J Osteoporosis Bone Miner Res.* (2018) 11:26–3.

[13] Kato H Ochiai-Shino H Onodera S Saito A Shibahara T Azuma T. Promoting effect of 1,25(OH)2 vitamin D3 in osteogenic differentiation from induced pluripotent stem cells to osteocyte-like cells. *Open Biol.* (2015) 5:140201. 10.1098/rsob.140201 25652541 PMC4345281

[14] Qian H Yu Y Wu Q Wang S Zhong C. Correlation analysis between bone mineral density and serum vitamin D IGF-1 IGFBP-3 in children with dwarfism. *Hebei Med*. (2022) 28:1148–52.

[15] Luo H Li C Jun M. Correlation study of height and serum 25-hydroxyvitamin D and IGF-1 levels in children aged 3-14 years old. *Mater Child Health Care China.* (2022) 37:24.

[16] Soliman A El Banna N Abdel FM ElZalabani M Ansari B. Bone mineral density in prepubertal children with beta-thalassemia: correlation with growth and hormonal data. *Metabolism.* (1998) 47:541–8. 10.1016/s0026-0495(98)90237-2 9591744

[17] Davies NM Holmes MV Davey Smith G. Reading Mendelian randomisation studies: a guide, glossary, and checklist for clinicians. *BMJ.* (2018) 362:k601. 10.1136/bmj.k601 30002074 PMC6041728

[18] Yang H Chen L Liu K Li C Li H Xiong K Mendelian randomization rules out the causal relationship between serum lipids and cholecystitis. *BMC Med Genomics.* (2021) 14:224. 10.1186/s12920-021-01082-y 34535143 PMC8447629

[19] Smith GD Ebrahim S. ‘Mendelian randomization’: can genetic epidemiology contribute to understanding environmental determinants of disease. *Int J Epidemiol.* (2003) 32:1–22. 10.1093/ije/dyg070 12689998

[20] Hemani G Zheng J Elsworth B Wade KH Haberland V Baird D The MR-Base platform supports systematic causal inference across the human phenome. *Elife.* (2018) 7:e34408. 10.7554/eLife.34408 29846171 PMC5976434

[21] Jiang X O’Reilly P Aschard H Hsu Y Richards J Dupuis J Genomewide association study in 79,366 European-ancestry individuals informs the genetic architecture of 25-hydroxyvitamin D levels. *Nat Commun.* (2018) 9:260. 10.1038/s41467-017-02662-2 29343764 PMC5772647

[22] Revez JA Lin T Qiao Z Xue A Holtz Y Zhu Z Genome-wide association study identifies 143 loci associated with 25 hydroxyvitamin D concentration. *Nat Commun.* (2020) 11:1647. 10.1038/s41467-020-15421-7 32242144 PMC7118120

[23] Chang CC Chow CC Tellier LC Vattikuti S Purcell SM Lee JJ. Second-generation PLINK: rising to the challenge of larger and richer datasets. *Gigascience.* (2015) 4:7. 10.1186/s13742-015-0047-8 25722852 PMC4342193

[24] Burgess S Butterworth A Thompson SG. Mendelian randomization analysis with multiple genetic variants using summarized data. *Genet Epidemiol.* (2013) 37:658–65. 10.1002/gepi.21758 24114802 PMC4377079

[25] Burgess S Small DS Thompson SG. A review of instrumental variable estimators for Mendelian randomization. *Stat Methods Med Res.* (2017) 26:2333–55. 10.1177/0962280215597579 26282889 PMC5642006

[26] Bowden J Davey Smith G Haycock PC Burgess S. Consistent estimation in Mendelian randomization with some invalid instruments using a weighted median estimator. *Genet Epidemiol.* (2016) 40:304–14. 10.1002/gepi.21965 27061298 PMC4849733

[27] Bowden J Davey Smith G Burgess S. Mendelian randomization with invalid instruments: effect estimation and bias detection through Egger regression. *Int J Epidemiol.* (2015) 44:512–25. 10.1093/ije/dyv080 26050253 PMC4469799

[28] Burgess S Thompson SG. Interpreting findings from Mendelian randomization using the MR-Egger method. *Eur J Epidemiol.* (2017) 32:377–89. 10.1007/s10654-017-0255-x 28527048 PMC5506233

[29] Bowden J Del Greco MF Minelli C Davey Smith G Sheehan NA Thompson JR. Assessing the suitability of summary data for two-sample Mendelian randomization analyses using MR-Egger regression: the role of the I2 statistic. *Int J Epidemiol.* (2016) 45:1961–74. 10.1093/ije/dyw220 27616674 PMC5446088

[30] Verbanck M Chen CY Neale B Do R. Detection of widespread horizontal pleiotropy in causal relationships inferred from Mendelian randomization between complex traits and diseases. *Nat Genet.* (2018) 50:693–8. 10.1038/s41588-018-0099-7 29686387 PMC6083837

[31] Bowden J Del Greco MF Minelli C Davey Smith G Sheehan NA Thompson JR. A framework for the investigation of pleiotropy in two-sample summary data Mendelian randomization. *Stat Med.* (2017) 36:1783–802. 10.1002/sim.7221 28114746 PMC5434863

[32] Kamenický P Mazziotti G Lombès M Giustina A Chanson P. Growth hormone, insulin-like growth factor-1, and the kidney: pathophysiological and clinical implications. *Endocr Rev.* (2014) 35:234–81. 10.1210/er.2013-1071 24423979

[33] Witkowska-Sędek E Kucharska A Rumińska M Pyrżak B. Relationship between 25(OH)D and IGF-I in children and adolescents with growth hormone deficiency. *Adv Exp Med Biol.* (2016) 912:43–9. 10.1007/5584_2016_212 26987336

[34] Song Y Kato S Fleet JC. Vitamin D receptor (VDR) knockout mice reveal VDR-independent regulation of intestinal calcium absorption and ECaC2 and calbindin D9k mRNA. *J Nutr.* (2003) 133:374–80. 10.1093/jn/133.2.374 12566470

[35] Forouhi N Luan J Cooper A Boucher B Wareham N. Baseline serum 25-hydroxy vitamin d is predictive of future glycemic status and insulin resistance: the medical research council ely prospective study 1990-2000. *Diabetes.* (2008) 57:2619–25. 10.2337/db08-0593 18591391 PMC2551670

[36] Hyppönen E Boucher B Berry D Power C. 25-hydroxyvitamin D, IGF-1, and metabolic syndrome at 45 years of age: a cross-sectional study in the 1958 British Birth Cohort. *Diabetes.* (2008) 57:298–305. 10.2337/db07-1122 18003755

[37] Ciresi A Giordano C. Vitamin D across growth hormone (GH) disorders: from GH deficiency to GH excess. *Growth Horm IGF Res.* (2017) 33:35–42. 10.1016/j.ghir.2017.02.002 28372721

[38] Ameri P Giusti A Boschetti M Murialdo G Minuto F Ferone D. Interactions between vitamin D and IGF-I: from physiology to clinical practice. *Clin Endocrinol.* (2013) 79:457–63. 10.1111/cen.12268 23789983

[39] Locatelli V Bianchi V. Effect of GH/IGF-1 on bone metabolism and osteoporsosis. *Int J Endocrinol.* (2014) 2014:235060. 10.1155/2014/235060 25147565 PMC4132406

[40] Varkaneh H Rinaldi G Hekmatdoost A Fatahi S Tan S Shadnoush M The influence of vitamin D supplementation on IGF-1 levels in humans: a systematic review and meta-analysis. *Ageing Res Rev.* (2019) 57:100996. 10.1016/j.arr.2019.100996 31816443

[41] Ameri P Giusti A Boschetti M Bovio M Teti C Leoncini G Vitamin D increases circulating IGF1 in adults: potential implication for the treatment of GH deficiency. *Eur J Endocrinol.* (2013) 169:767–72. 10.1530/EJE-13-0510 24005315

[42] Crew K Anderson G Hershman D Terry M Tehranifar P Lew D Randomized double-blind placebo-controlled biomarker modulation study of vitamin D supplementation in premenopausal women at high risk for breast cancer (SWOG S0812). *Cancer Prev Res.* (2019) 12:481–90. 10.1158/1940-6207 PMC6609474 31138522

[43] Kamycheva E Berg V Jorde R. Insulin-like growth factor I, growth hormone, and insulin sensitivity: the effects of a one-year cholecalciferol supplementation in middle-aged overweight and obese subjects. *Endocrine.* (2013) 43:412–8. 10.1007/s12020-012-9825-6 23109222

[44] Mortensen C Molgaard C Hauger H Kristensen M Damsgaard CT. Winter vitamin D3 supplementation does not increase muscle strength, but modulates the IGF-axis in young children. *Eur J Nutr.* (2019) 58:1183–92. 10.1007/s00394-018-1637-x 29450728

[45] Trummer C Schwetz V Pandis M Grubler MR Verheyen N Gaksch M Effects of vitamin D supplementation on IGF-1 and calcitriol: a randomized controlled trial. *Nutrients.* (2017) 9:623. 10.3390/nu9060623 28629132 PMC5490602

[46] Burgess S. Sample size and power calculations in Mendelian randomization with a single instrumental variable and a binary outcome. *Int J Epidemiol.* (2014) 43:922–9. 10.1093/ije/dyu005 24608958 PMC4052137

[47] Brabant G Wallaschofski H. Normal levels of serum IGF-I: determinants and validity of current reference ranges. *Pituitary.* (2007) 10:129–33. 10.1007/s11102-007-0035-9 17487588

[48] Neale Lab (2018). Available online at: www.nealelab.is/uk-biobank

