# Supplementary material for: Causal associations between insulin-like growth factor 1 and vitamin D levels: a two-sample bidirectional Mendelian randomization study
Source: Front Nutr. 2023 May 17;10:1162442. doi: 10.3389/fnut.2023.1162442 (PMC10229803; doi:10.3389/fnut.2023.1162442)
Supplement: Supplementary file 1 [file Presentation_1.pdf]

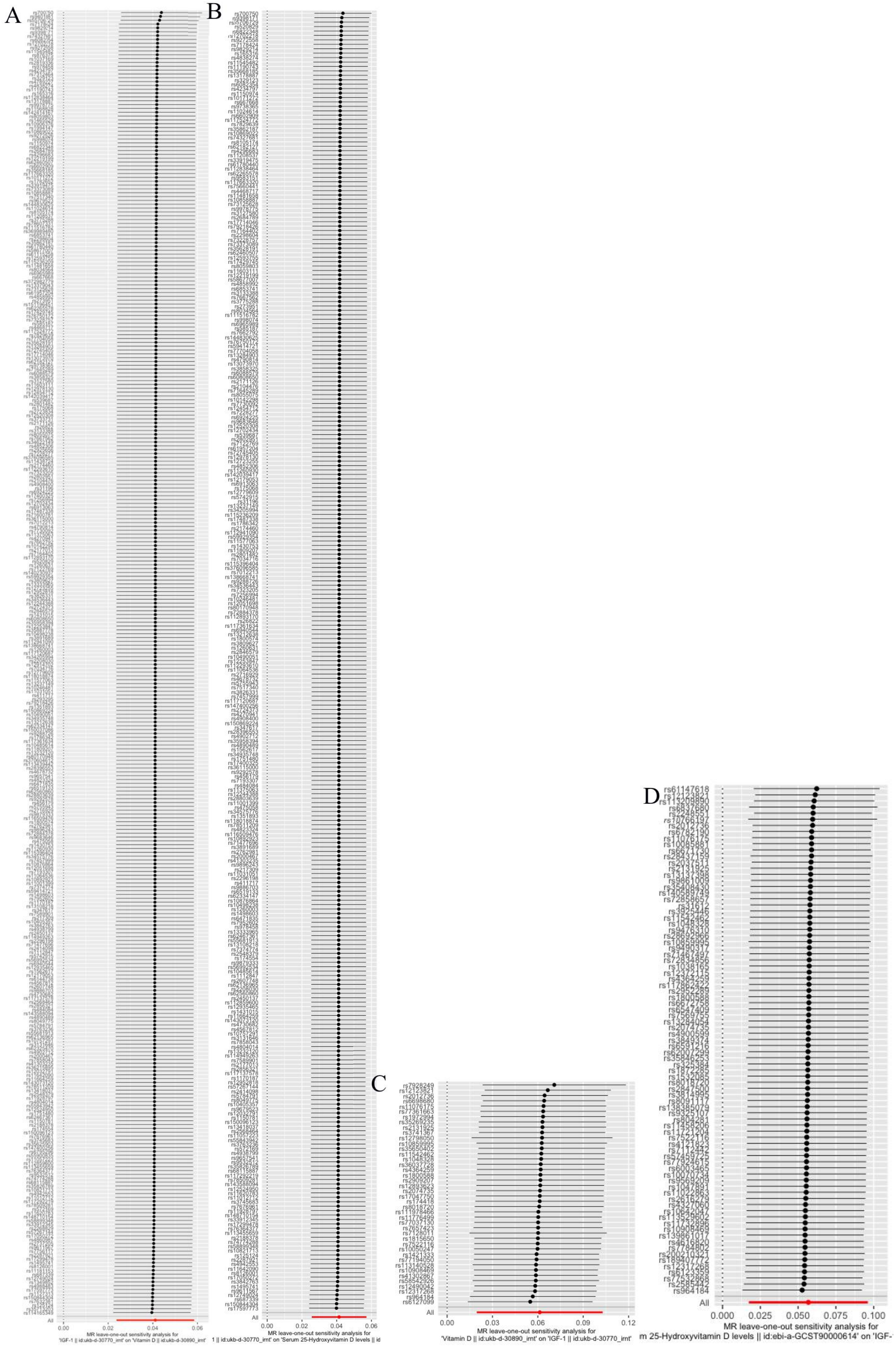
A
B
C
D
All
0.00
0.02
0.04
0.06
MR leave-one-out sensitivity analysis for
'IGF-1 || id:ukb-d-30770_irnt' on 'Vitamin D || id:ukb-d-30890_irnt'
MR leave-one-out sensitivity analysis for
1 || id:ukb-d-30770_irnt' on 'Serum 25-Hydroxyvitamin D levels || id
0.03
0.06
0.09
0.12
MR leave-one-out sensitivity analysis for
'Vitamin D || id:ukb-d-30890_irnt' on 'IGF-1 || id:ukb-d-30770_irnt'
0.000
0.025
0.050
0.075
0.100
MR leave-one-out sensitivity analysis for
m 25-Hydroxyvitamin D levels || id:ebi-a-GCST90000614' on 'IGF-

Supplement figure 1: MR leave-one-out showing the sensitivity analysis of exposure on outcome. (A) The effect of IGF-1 on vitamin D. (B) The effect of IGF-1 on 25-hydroxyvitamin D. (C) The effect of vitamin D on IGF-1. (D) The effect of 25-hydroxyvitamin D on IGF-1.
